# Class I PI3Ks in arterial thrombosis

**DOI:** 10.18632/aging.101841

**Published:** 2019-02-24

**Authors:** Pierre-Alexandre Laurent, Bernard Payrastre, Marie-Pierre Gratacap

**Affiliations:** 1 Institute of Cadiovascular and Metabolic Diseases, UMR 1048, University Toulouse III, Toulouse, France

**Keywords:** thrombosis, platelet, PI3K

Myocardial infarction and strokes, which are caused by arterial thrombosis in heart and brain respectively, are the most frequent causes of mortality and morbidity in the western world. Several clinical trials have demonstrated the efficiency of anti-thrombotic agents targeting platelets (aspirin, P2Y12-antagonist, anti-GPIIbIIIa) in the treatment and the prevention of arterial thromboembolism [1]. However, these approaches can lead to bleeding complications or can be insufficiently effective due to drug resistance mechanisms. Besides their critical role in hemostasis and thrombosis, platelets are also implicated in wound healing, angiogenesis and inflammation through the secretion of highly active compounds such as growth factors, cytokines or bioactive lipids.

There are several risk factors for arterial thromboembolism such as advanced age, hypertension, obesity, metabolic syndrome and type 2 diabetes which is continuously increasing all over the world and a major risk factor for cardiovascular diseases. Many studies have reported platelet hyperactivation in patients with metabolic syndrome and/or diabetes. Associated with a disturbed vascular endothelium layer, platelet hyperaggregability is a predisposing factor for arterial thrombosis. In addition, diabetes is frequently associated with a lower response to antiplatelet drugs. The molecular mechanisms implicated are not yet fully understood. Several studies also point to a critical role of platelets in the progression of reperfusion injury following ischemic stroke. During this process, platelet adhesion to activated endothelial cells appears to be critically involved, unlike platelet aggregation which inhibition has been shown to increases the risk of intracranial hemorrhage and mortality, reducing the benefit-risk ratio. In contrast, preventing platelet adhesion to the damaged vessel wall by blocking platelet surface adhesion receptors particularly GPVI or GPIbα has been found to offer a significant degree of neuroprotection in experimental stroke models without increasing the frequency of intracranial hemorrhage.

Hence it’s becoming clear that a better understanding of the molecular mechanisms driving platelet responses to environment cues is crucial to discover new pharmacological targets and to develop novel therapeutic strategies to face cardiovascular diseases.

Class I phosphoinositide 3-kinases (PI3Ks) are lipid kinases that catalyze the phosphorylation of the 3-position of the inositol ring of phosphatidylinositol(4,5)bisphosphate (PtdIns(4,5)P_2_) to produce the lipid second messenger PtdIns(3,4,5)P_3_ that is able to recruit and activate a wide range of signaling proteins, including Akt. By interacting with specific protein domains, phosphoinositides play a pivotal role in the spatio-temporal organization of cell signaling, membrane remodeling, intracellular trafficking and cytoskeletal dynamics. Class I PI3Ks have been extensively studied in different models in the last decades. Their critical roles in diseases, particularly in cancers, have stimulated the development of isoform-specific inhibitors. Some of these inhibitors are undergoing clinical trials for the treatment of solid cancer and overgrowth syndrome with gain-of-function mutations of the PI3KCA gene (PI3Kα). Moreover, the selective PI3Kδ inhibitor idelalisib has received FDA approval for the treatment of B-cell malignancies. The use of class I PI3Ks inhibitors has also been proposed for the treatment of immune diseases including arthritis and PI3Kδ-selective inhibitors are currently being explored in the treatment of inflammatory diseases.

What is the impact of class I PI3Ks inhibitors in platelets activation and thrombosis?

Although all class I PI3Ks isoforms are expressed in platelets, class I PI3Kβ has a major role and has been proposed as a potential antithrombotic drug target. By generating mice with a selective inactivation of PI3Kβ in the megakaryocyte lineage, we [2] and others [3,4] have demonstrated that this lipid kinase is involved downstream of the main platelet receptors with a strong contribution of the Gi coupled receptor for ADP, P2Y12. While platelet PI3Kβ is dispensable for thrombus growth and stability at normal arterial shear, it is essential to preserve thrombus integrity at high shear rate encountered in stenosed arteries. Under stringent hemodynamic forces, lack of PI3Kβ appears to affect platelet-platelet interaction within the thrombi rather than the platelet-matrix interaction. Since its inhibition spares primary hemostasis, selective PI3Kβ inhibitors remain of potential interest as new target for anti-thrombotic drugs. However, it will be important to determine whether unsafe platelet emboli released from the growing thrombus on treatment with PI3Kβ inhibitors may be prevented by association with aspirin.

Regarding the other class I PI3Ks, mice deficient for the PI3Kγ isoform are resistant to experimental thromboembolism induced by ADP exclusively, whereas PI3Kδ has no significant role in platelets. We recently generated a mouse model with class I PI3Kα deficient platelets [5] and observed that absence or pharmacological inhibition of PI3Kα had not significant impact on primary hemostasis. Compared to anti-platelet drugs, such as clopidogrel or GPIIbIIIa blockers, PI3Kα invalidation or inhibition had a much weaker impact on thrombus formation *in vivo*. These results suggest that PI3Kα inhibitors have a weak antithrombotic effect and, on their own, are unlikely to increase the bleeding risk. This is important information for cancer patients treated with these inhibitors. However, the impact on the bleeding risk of PI3Kα inhibitors in association with anti-platelet drugs or in patients with thrombocytopenia remains to be investigated. From a mechanical point of view, PI3Kα and PI3Kβ are not redundant but complementary in platelets [6–8]. PI3Kα is important to prime platelets following low-level activation of tyrosine kinase-dependent pathways. Under these conditions, PI3Kα contributes to ADP secretion which is then essential for a full activation of PI3Kβ, the major producer of PtdIns(3,4,5)P_3_ and Akt activator [5].

Thus, among class I PI3Ks, PI3Kβ is a potential antithrombotic drug target to prevent the formation of occlusive arterial thrombus; whether it is also a target to improve ischemic-reperfusion injury in stroke remains to be established.
